# In Vivo Visualization and Quantification of Neutrophil Elastase in Lungs of COVID-19 Patients: A First-in-Humans PET Study with ^11^C-NES

**DOI:** 10.2967/jnumed.122.263974

**Published:** 2023-01

**Authors:** Gunnar Antoni, Mark Lubberink, Jens Sörensen, Elin Lindström, Mathias Elgland, Olof Eriksson, Michael Hultström, Robert Frithiof, Anders Wanhainen, Jonathan Sigfridsson, Paul Skorup, Miklos Lipcsey

**Affiliations:** 1 Department of Medicinal Chemistry, Uppsala University, Uppsala, Sweden;; 2 Department of Surgical Sciences, Uppsala University, Uppsala, Sweden; and; 3 Department of Medicinal Sciences, Uppsala University, Uppsala, Sweden

**Keywords:** neutrophil elastase, PET, ^11^C-GW457427

## Abstract

Coronavirus disease 2019 (COVID-19) can cause life-threatening lung inflammation that is thought to be mediated by neutrophils. The aim of the present work was to evaluate a novel PET tracer for neutrophil elastase (NE). **Methods:** In this first-in-humans study, 4 patients with hypoxia due to COVID-19 and 2 healthy controls were investigated with PET using ^11^C-NES and ^15^O-water for visualization and quantification of NE and perfusion in the lungs, respectively. **Results:** ^11^C-NES accumulated selectively in lung areas with COVID-19 opacities on CT scans, suggesting high levels of NE there. In the same areas, perfusion was severely reduced in comparison to healthy lung tissue as measured with ^15^O-water. **Conclusion:** The data suggest that NE is associated with severe lung inflammation in COVID-19 patients and that inhibition of NE could potentially reduce the acute inflammatory process and improve the condition.

## Visual Abstract

**Figure:**
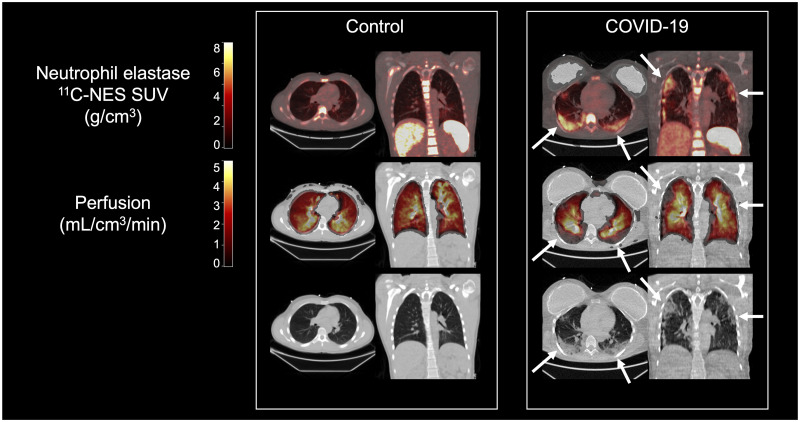

A significant number of patients with coronavirus disease 2019 (COVID-19), caused by the severe acute respiratory syndrome coronavirus 2, present with respiratory failure due to lung inflammation. This process has been postulated to involve lung infiltration of neutrophil granulocytes, based on postmortem histology and neutrophil extracellular trap (NET) markers (1). Recent data have shown that NETs also promote thrombosis (2). Neutrophil elastase (NE), a protease found in neutrophil granulocytes, is part of the innate immune response but could mediate the lung injury seen in COVID-19 (3–8). To date, in vivo studies of NE in the lungs of COVID-19 patients have not been possible. However, the development of the PET tracer ^11^C-NES ((3S,3aS,6aR)-4-(5-((cyclopropylamino)methyl)pyrazine-2-carbonyl)-3-isopropyl-1-(methylsulfonyl)hexahydropyrrolo[3,2-b]pyrrol-2(1H)-one, or ^11^C-GW457427) has made detection of NE possible in the clinical setting. ^11^C-NES has been extensively studied in animal models for lung inflammation, and binding specificity has been verified in blocking studies with NE inhibitors (9). It measures both extracellular and intracellular NE.

We hypothesized that COVID-19 patients would have significant amounts of NE in inflamed lung areas. Therefore, we conducted a first-in-humans study with ^11^C-NES combining PET and CT in 4 patients and 2 healthy controls. Additionally, we assessed perfusion in the patients’ lungs with ^15^O-water. Low-dose CT was used for attenuation correction and visualization of radiologic findings such as ground-glass opacity (GGO) and consolidated areas in the lungs, characteristic of COVID-19 patients (10).

## MATERIALS AND METHODS

### Subjects

This study was approved by the Swedish Medical Products Agency (EudraCT 2020-003980-24) and the Swedish Ethical Review Authority (*Diarienummer* 2020-07017). The Helsinki Declaration and its subsequent revisions were observed. We recruited 4 symptomatic and hospitalized patients, at least one of either sex, who had polymerase chain reaction–verified COVID-19 and had been admitted to the hospital because of hypoxic respiratory failure and treated with supplemental oxygen at a non–intensive-care unit. We also recruited 2 healthy controls, in accordance with the approved procedure in the ethical permit. The characteristics of the patients and healthy controls are presented in Table 1 and Supplemental Table 1 (supplemental materials are available at http://jnm.snmjournals.org).

**TABLE 1.** Subject Characteristics

Characteristic	Patient 1	Patient 2	Patient 3	Patient 4	Control 1	Control 2
Body mass index (kg/m^2^)	25	26	30	32	23	30
Vaccinated against COVID-19	No	No	No	No	Yes	Yes
Symptom duration (d)	20	21	12	13	NA	NA
Admission duration at PET scan (d)	2	11	1	3	NA	NA
SARS-CoV-2 RNA test*	Positive	Positive	Positive	Positive	Negative	Negative
Virus variant	No data	Delta	Delta	Delta	NA	NA
Viremia SARS-CoV-2 RNA serum^†^	Neg	Neg	36	Neg	NA	NA
C-reactive protein (mg/L)	31	19	259	8	<0.3	19
Peak C-reactive protein (mg/L)	31	396	259	115	NA	NA
White blood cell count (10^9^/mL)	8.9	7.1	5.2	6.5	4	NA
Lymphocyte count (10^9^/mL)	1.3	0.6	0.4	1.3	1.1	NA
Procalcitonin (mg/L)	0.03	0.27	0.05	0.04	<0.02	<0.02
Ferritin (μg/L)	209	1.255	301	2.423	58	48
Lactate dehydrogenase (μkat/L)	5.3	4.6	4.9	6.1	2.8	2
d-dimer (mg/L)	0.6	4.4	1.4	0.3	<0.3	<0.3
Peak oxygen administered (L/min)^‡^	2	15	1	5	NA	NA

* Nasopharynx and throat.

^†^ Cycle threshold value N gene/negative.

^‡^ Peak oxygen delivery to maintain saturation of 93%–96% during hospital stay.

NA = not applicable; SARS-CoV-2 = severe acute respiratory syndrome coronavirus 2.

Median age was 48 y (range, 24–57 y) for patients 1–4 and 26 y (range, 22–29 y) for controls 1 and 2 (age and sex are not shown because of anonymization).

Verbal and written informed consent was obtained from the participants.

### Tracer Production

^11^C-NES was produced in accordance with good manufacturing practices, and its safety was investigated in a microdosing toxicology study (9).

### Safety

^11^C-NES was used for the first time in humans. Before and after PET, all subjects were examined for vital signs, phlebitis, and rash and underwent electrocardiography. No subject showed any adverse effects after being administrated approximately 5 MBq/kg of body weight, corresponding to a total ^11^C-NES amount of about 5 μg.

### PET Scanning

All subjects were scanned on a digital time-of-flight PET/CT system (Discovery MI-5; GE Healthcare) with a 25-cm axial field of view. The subjects were supine during the entire examination. After a low-dose CT scan for anatomic localization and attenuation correction, a 4-min dynamic PET scan was started simultaneously with administration of a controlled bolus injection of 400 MBq of ^15^O-water (5 mL at 1 mL/min followed by 35 mL of saline at 2 mL/min). At least 15 min later, a second dynamic PET scan was started simultaneously with administration of a 5 MBq/kg controlled bolus injection of ^11^C-NES (range, 4.2–5.3 MBq/kg), with a duration of 15 min in healthy controls and 60 min in COVID-19 patients. List-mode data were rebinned into frames of increasing durations over the course of the scan (1 × 10, 8 × 5, 4 × 10, 2 × 15, 3 × 20, and 2 × 30 s for ^15^O-water and 1 × 10, 8 × 5, 4 × 10, 3 × 20, 2 × 30, and 5 × 60 s for ^11^C-NES in all subjects, followed by 4 × 300 and 3 × 600 s in patients). Dynamic images were reconstructed using time-of-flight ordered-subset expectation maximization, including resolution recovery, with 3 iterations, 16 subsets, and a 5-mm gaussian postprocessing filter. All appropriate corrections for accurate quantification were applied. A whole-body low-dose CT and PET scan of ^11^C-NES (3 min per bed position) was acquired at approximately 1 h after injection and reconstructed using block-sequential regularized expectation maximization (Q.Clear; GE Healthcare) with a regularization parameter of 500.

### Pulmonary Perfusion

For calculation of lung perfusion based on the ^15^O-water PET data, systemic and pulmonary circulation blood time–activity curves were determined using automated cluster analysis in aQuant Research software (Medtrace Pharma A/S). Lung perfusion images were calculated by implementing a nonnegative least-squares basis function in a single-tissue-compartment model in which the pulmonary circulation blood curve was the input function, with fitted pulmonary and systemic blood volume parameters. Only voxels with density of less than 0.9 g/cm^3^, based on the low-dose CT images, were included in the calculations. Pulmonary perfusion was calculated as the influx rate of ^15^O-water. Volumes of interest were drawn on pulmonary perfusion images over representative parts of normal-appearing and GGO and consolidated lung tissue based on the CT images.

### ^11^C-NES Data Analysis

Volumes of interest were drawn on summed ^11^C-NES images as above and transferred to the dynamic PET images to obtain time–activity curves. ^11^C-NES SUVs were calculated as radioactivity concentrations normalized to injected activity per kilogram of body weight.

## RESULTS

^11^C-NES was obtained with a radiochemical purity higher than 98% and a molar activity in the range of 180–330 GBq/μmol. Chemical purity was high, with no traces of the precursor GW611437.

**FIGURE 1.**
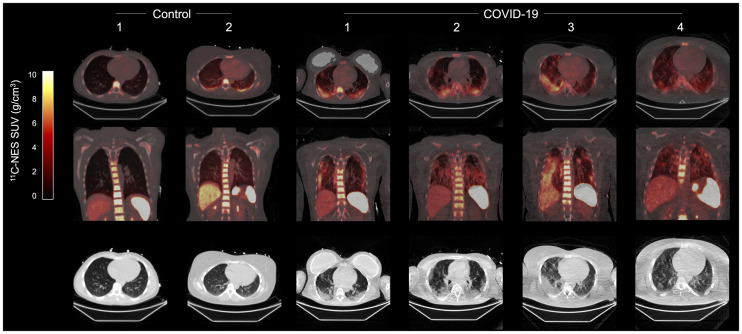
PET/CT images of 4 patients and 2 controls (3 male, 3 female) at 1 h after injection. Upper 2 rows are ^11^C-NES images; lower row is CT images.

In all patients, ^11^C-NES accumulated in the areas of the lungs with GGO and consolidations identified on CT (Fig. 1) and low perfusion as measured with ^15^O-water.

The very low nonspecific signal of ^11^C-NES in nontarget areas resulted in a high target-to-background ratio. SUVs for liver and spleen are presented in Supplemental Table 2.

**FIGURE 2.**
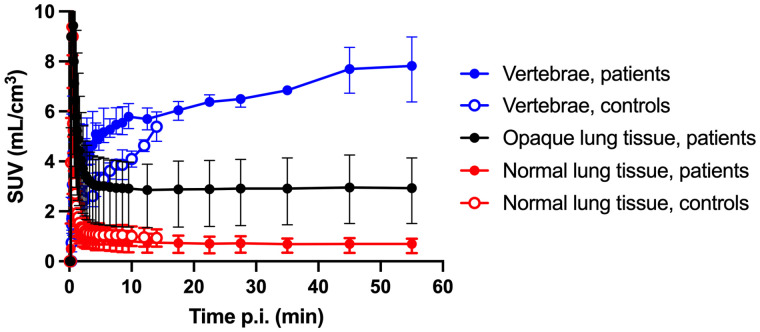
SUV of ^11^C-NES vs. time in GGO and consolidations and normal lung tissue in COVID-19 patients (*n* = 4) and in lung tissue in healthy controls (*n* = 2). SUVs in vertebrae in patients and controls are shown for comparison. Error bars denote range of values. p.i. = after injection.

Lung uptake of ^11^C-NES had already plateaued at 10 min after injection (Fig. 2), indicating retention of tracer bound to NE. ^11^C-NES uptake in the vertebrae increased over time, with a significantly faster initial phase in patients than in controls.

**FIGURE 3.**
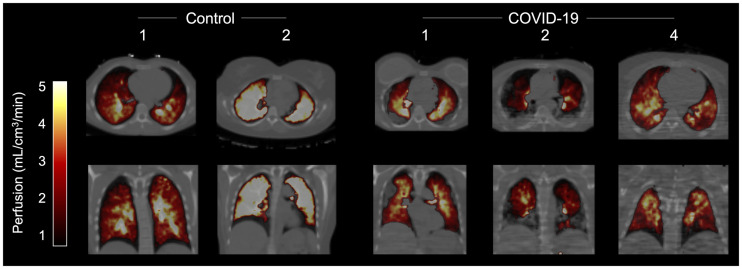
^15^O-water perfusion PET/CT images of 3 patients and 2 controls.

The lung perfusion as measured with ^15^O-water showed a clear reduction in GGO and consolidated areas compared with healthy lung tissue (Fig. 3).

The SUV data from ^11^C-NES and the absolute values of perfusion are summarized in Table 2.

**TABLE 2.** SUV for ^11^C-NES and Perfusion Data

PET data	Patient 1	Patient 2	Patient 3	Patient 4	Control 1	Control 2
^11^C-NES in healthy parts of lungs (SUV)	0.8	0.3	1.1	NA*	0.6	1.3
^11^C-NES in opaque parts of lungs (SUV)	3.9	1.3	2.4	3.8	NA	NA
Perfusion in healthy parts of lungs (mL/cm^3^/min)	2.7	1.5	NA^†^	2.6	2.6	3.9
Perfusion in opaque parts of lungs (mL/cm^3^/min)	1.9	0.7	NA^†^	1.5	NA	NA

* No healthy parts of lung could be identified.

^†^ Because of technical problems, no ^15^O-water PET scan was performed.

NA = not applicable; opaque = radiographic opacities such as GGO or consolidations.

## DISCUSSION

All patients in this study were hospitalized with severe COVID-19 and respiratory failure and clinically manifested severe lung inflammation without signs of bacterial superinfections. Since the patients were in the second to third week of infection, that is, a late stage, the lung symptoms and low blood oxygenation levels for patients breathing normal air without added oxygen indicated extensive inflammation, not the effect of an ongoing virus infection. The high accumulation of ^11^C-NES in bone marrow and spleen (Fig. 1) can be explained by its binding to NE in resident neutrophils. The accumulation of ^11^C-NES was considerably higher in areas with GGO and consolidations than in healthy lung tissue, whether in the same patient or in healthy controls. The nearly perfect anatomic match between COVID-19 opacities and the accumulation of ^11^C-NES, together with the high uptake in the vertebrae (which are rich in bone marrow) and in the spleen, strongly indicate that tracer uptake is a measure of NE. ^11^C-NES is also taken up by neutrophil granulocytes in blood, and uptake in healthy lung tissue reflects mainly the blood-borne radioactivity, explaining the low variability. A uniform gradient of ^11^C-NES uptake in the direction of gravity was seen in one healthy control. In contrast, the uptake in inflamed areas is due to accumulation of neutrophil granulocytes and thus a measure of immune-mediated inflammation. Lastly, the GGO and consolidations might also be explained by formation of NETs in peripheral small blood vessels. This possibility is supported by the identification of NETs in postmortem tissue from COVID-19 patients, suggesting that the uptake of ^11^C-NES could partly be related to binding to elastase deposited on DNA strands in NETs (2,4,11,12).

The inflamed areas, especially consolidations, had much lower perfusion than the radiologically healthy lung tissue, potentially representing hypoxic vasoconstriction. As some perfusion remained in the inflamed areas, shunting of blood may have contributed to the hypoxia seen in those patients. Alternatively, entrapment of aggregated activated neutrophils in the capillary beds, leading to microthrombosis, could have contributed to the reduction in blood flow measured in the affected parts of the lungs. This results in an elastase-mediated destruction of tissue, creating a concomitant mismatch in ventilation–perfusion and affecting blood oxygenation negatively.

This study was limited by the few patients investigated and was therefore of a descriptive nature, without statistical measures.

## CONCLUSION

In this first-in-humans study with the selective and specific NE PET tracer ^11^C-NES, we demonstrated high accumulation in lung areas with low perfusion and COVID-19 opacities as measured with ^15^O-water and CT, respectively. This finding can be interpreted as a sign of neutrophil-mediated pulmonary inflammation affecting lung function and can partly explain the late-stage conditions of COVID-19 patients. Lung perfusion measurements suggest that there is hypoxic vasoconstriction in areas with ongoing inflammation. Moreover, data suggest that NE might be involved in the severe lung inflammation.

## DISCLOSURE

This study was financially supported by Uppsala University ALF project 1011799. No other potential conflict of interest relevant to this article was reported.

## Notes

KEY POINTS **QUESTION:** Are neutrophils associated with the severe lung inflammation in COVID-19 patients, and do areas of increased uptake of ^11^C-NES correlate with COVID-19 opacities and reduced perfusion, as measured with CT and ^15^O-water, respectively? **PERTINENT FINDINGS:** Uptake of ^11^C-NES was found in the same lung areas as COVID-19 opacities and low perfusion. A clear difference was seen between COVID-19 patients and healthy controls. **IMPLICATIONS FOR PATIENT CARE:** It may be possible to treat the acute neutrophil-mediated lung inflammation with elastase inhibitors and by interfering with the cytokine/chemokine signaling pathways responsible for recruiting neutrophils.
